# Neuropeptide Profiles of Mammalian Male Genital Tract: Distribution and Functional Relevance in Reproduction

**DOI:** 10.3389/fvets.2022.842515

**Published:** 2022-03-30

**Authors:** Jamiu O. Omirinde, Idris A. Azeez

**Affiliations:** Department of Veterinary Anatomy, Faculty of Veterinary Medicine, University of Jos, Jos, Nigeria

**Keywords:** neuropeptides, nerve fibers, mammals, male genitalia, accessory sex glands, reproduction

## Abstract

Neuropeptides are secretory peptides characterized by small chains of amino acids linked by peptide bonds. They are majorly found in some mammalian neurons and glial cells, where they modulate a variety of physiological homeostasis. In the male genital tract, they are mostly found in the neuronal fibers supplying the vasculature, smooth muscle layer, interstitium, and *lamina propria* of the tunica mucosa of the various reproductive organs. Functionally, neuropeptides are strongly implicated in vascular temperature regulations, spermatozoa extrusion, epididymal content transportation, and movement of accessory gland secretions. This review provides an overview of neuropeptides with respect to their synthesis, release, and mechanism of actions, with emphasis on the locally acting neuropeptides, such as substance P (SP), neuropeptide Y (NPY), vasoactive intestinal peptides (VIP), calcitonin gene-related peptide (CGRP), galanin (GAL), cholecystokinin (CCK), C-terminal flanking peptide of NPY (CPON), peptide histidine isoleucine (PHI), and met- and leu-enkephalins (M-ENK and L-ENK) along the male genital tract (i.e., the spermatic cord, testis, epididymis, ductus deferens, and accessory sex organs) of 14 species of mammals and their marked influence on reproduction. This review also revealed from documented reports that the vast majority of neuropeptides present in the autonomic nerve supply to the male genital tract probably coexist with other peptides or with various neurotransmitters (tyrosine hydroxylase, dopamine beta hydroxylase, and 5-hydroxytryptamine). In addition, documented evidence of variation in age, season, and intraspecies differences were identified as notable factors of influence in peptidergic nerve fiber distribution.

## Introduction

According to Burbach et al. (1), neuropeptides (NPs) are minute protein-rich substances, which are secreted and released by neuronal cells, *via* regulated secretory routes. They act on neural substrates. Of note, distinction exists between neuropeptides and other peptides (especially peptide hormones) in that it is strictly neurons that secrete neuropeptides, while peptide hormones found their origin in the periphery (i.e., outside of the CNS). However, the same sets of regulatory enzymes control the synthesis, modification, and biodegradation of both peptides. Moreover, neuropeptides could exert autocrine, paracrine, and endocrine functions, just like peptide hormones (1).

In contrast with neurotransmitters, the absence of peptide reuptakes, and the need for volume transmission (non-synaptic dispersion) culminates in the relatively long-lived impacts of neuropeptides. Their actions are enhanced by G-protein-coupled receptors (2). Neuropeptides perform neuromodulatory roles in the central nervous system (CNS), while they act as signaling molecules in the periphery. Additionally, the high ligand affinities of NPs allow the minutest amounts of the peptides to still activate receptors (2).

There is great diversity in the functioning of neuropeptides, and this is highly due to the distinctions in the proteolysis of their precursors, primary sequence, covalent modifications, and length of the peptide (3). The bioactivities of neuropeptides (pre- and posttranslational modifications) are defined by their primary amino acid sequences (4). In neuroendocrine systems, neuropeptides are involved in the coordination of various cell–cell signaling mechanisms (3, 5), and their total involvements in intercellular signaling underscore the critical need to document the critical roles of these peptides in regulating specific physiological organ systems, for instance, the genital organs.

The mammalian male genitalia consist of organs that are involved in the development, maturation, transportation, and deposition of the male gametes (spermatozoa). By virtue of this, the male genital organs encompass the paired testes, the convoluted duct of epididymis, the deferent duct, the urethra, and the accessory glands (6). Many peptides, including neuropeptides, are synthesized and bound in the vertebrate gonads, and the peripheral hormones that bind to the gonads exert great influence on the gonadal neuropeptides. However, the neuropeptide profiles of the gonads are not well-understood either in isolation or in combination with other systemic neuropeptides (7).

The focus of this review is to profile the neuropeptides central to the morphophysiological functioning of the vertebrate mammal male genital tract, their distribution, and the variability of their expressions.

## Autonomic Innervation of the Mammalian Male Genitalia

The main part of the male genitalia, the testis, is innervated by complex networks of peripheral nerve [the superior and the inferior spermatic nerve (SSN and ISN)] fibers originating from the cranial and caudal mesenteric ganglia (these nomenclatures are homologous to the superior and inferior mesenteric ganglia in primates) (8, 9). The superior spermatic nerve (SSN) fiber chiefly derived its fibers from the cranial mesenteric ganglion together with inputs from the renal and aortic plexuses and, thereafter, continues to meet the testis along with testicular artery to penetrate the testis at the cranial pole (10). The SSN receives both the afferent and vagal parasympathetic fibers, while the inferior spermatic nerve (ISN) fibers are predominantly sympathetic (11, 12).

The ISN fiber leaves from both the caudal mesenteric ganglion and pelvic plexus together with the vas deferens and then access the caudal pole of the testes through the proper ligament of the tail of epididymis (13, 14). The accessory organs primarily receive dense autonomic innervation from the pelvic plexus situated close to many of the pelvic organs (15, 16). The neurons of the pelvic plexus in several species are made up of a mixture of both sympathetic and parasympathetic types, and receive preganglionic fibers coursing within hypogastric or pelvic nerves (17).

The rest of the genital tract receives innervation from the lumbosacral plexuses, which are made up of the genitofemoral (inguinal), pudendal, and in some species (e.g., man and equine), the ilioinguinal nerves (18). The genitofemoral nerve supplies the cremaster muscle and scrotal skin (18, 19). The pudendal nerve branches into the motor and sensory divisions at the level of the ischium. The somatosensory division innervates the genitalia, specifically the penis, scrotum, and perineum (20), while the motor division extends somatic fibers to the striated penile muscles. The two divisions unitedly carry efferent sympathetic fibers (involuntary motor) to the bulbospongiosus and ischiocavernosus muscles (20, 21).

## Nerve Fiber Chemical Coding

One of the research paths on the innervation of the reproductive tract in many species of animals studied dealt with the distribution and chemical coding of nerve fibers supplying the male genital organs (22). Several workers have optimized the use of the general and specific neuronal markers to determine nerve fiber presence, distribution, and chemical coding. Some of the general neuronal markers earlier used include specific-protein gene product-9.5 (PGP 9.5), neuron-specific enolase (NSE), growth-associated protein-43 (GAP 43), neurofilament (NF-70, 160, 200 kDa subunit proteins), synaptosomal-associated protein of 25 kDa (SNAP 25), and nerve growth factors p75 (NGF p75) (23–26). Meanwhile, the specific markers previously utilized for unraveling nerve fiber composition consists of cathecholaminergic [tyrosine hydroxylase (TH) and dopamine beta hydroxylase (DBH)], serotonergic [5-hydroxytryptamine (5-HT)], and peptidergic [substance P (SP), neuropeptide Y (NPY), vasoactive intestinal peptides (VIP), calcitonin gene-related peptide (CGRP), galanin (GAL), cholecystokinin (CCK), C-terminal flanking peptide of NPY (CPON), peptide histidine isoleucine (PHI), and met- and leu-enkephalin (M-ENK and L-ENK)] neuronal markers (23–25, 27).

The abovenamed sets of neuronal markers have been used to localize testicular nerves in the testicular capsules, around branches of the testicular artery, within interstitial Leydig cells, seminiferous tubular interstices, epididymal tubular interstitium, epididymal ductal muscular coat, and in the wall and parenchyma stroma of accessory glands in the Wistar rat, dog, bovine, pig, donkey, monkey, deer, man, camel, giant rat, house musk shrew, and African greater cane rats (23–32). It would be of great relevance to have a concise documentation on the findings (both recent and old) on the above neuropeptides and their distribution patterns in the genitalia of various animal species. The details about these are provided in the section below.

## Neuropeptide Localization and Distribution in the Male Genital Tract

We hereby provide an overview of the documented findings on neuropeptide localizations in the male genital tracts of the different mammalian species.

### Human Testis and Epididymis

Immunohistochemical technique has been used to localize neuropeptides in the testes of different age categories of man (subadult and adult) (27, 33, 34). The studies revealed the localization of NPY-immunopositive nerve fibers in the testicular capsule, peritubular myoid cells, in the Leydig cell, and seminiferous tubular interstices (particularly around the interstitial blood vessels). The VIP-positive nerve axons were demonstrated around intratesticular arterioles and Leydig cells (27, 33). For CGRP neuropeptide, positive sporadic demonstration was observed within the testicular capsule and was immuno-negative in the seminiferous tubular interstices. Substance P localization and distribution in the testes were found to be occasional and with many conflicting reports of presence or absence (27, 33, 34). Gong et al. (27) reported that the distribution as well as localization of CPON (C-terminal flanking peptide of NPY) in human testis was similar to the profile described for NPY. The M-ENK-positive nerve axons were exclusively demonstrated in the testicular capsule, while the PHI and GAL were immunonegative in the testis of man (33).

Taino (35) submitted that the peptidergic innervation of the epididymis in man was denser than its testicular counterpart. The study showed that strong NPY immunopositive axons were restricted to the epididymal tubular interstices and around the blood vessels, while the VIP, CGRP, PHI, and GAL positive nerve fibers were strongly immunolocalized only in the epididymal interstices. Except NPY nerve fibers, every other nerve fiber demonstrated in the epididymal interstitium in the study were varicose and not plexus by nature.

### Bovine Testis and Associated Structures

The comparative neuropeptide distribution in different age groups of bovine (calf: 5 weeks, subadult: 2 years and adult: 6–7 years) was investigated by Wrobel and Abu-Ghali (23). The study revealed that neuropeptide Y-positive nerve fibers were localized in the *tunica albuginea, mediastinum*, perimediastinal, and capsular arteries of the testis. In terms of contribution, about 50% of funicular, mesochial, and caudal nerve access to the testis were found to be NPY positive in the adult and were not expressed in the *septula testis*. While in the young calf and subadult bovine, the NPY nerve fibers from the three testicular nervous access constituted only about 33%. In addition, NPY nerve fibers were demonstrable in the *septula testis* of the younger age groups. Concerning the VIP nerve-positive axons, the authors observed immunoreactivity in the tunica albuginea, ligamentum propria, and caudal mesodeferens. The demonstration of VIP-positive nerve fibers in the testis was directly related to age [numerous in 5 weeks calf, less frequent in the subadult (2 years old) and absent in the adult bovine (6–7 yearrs)]. The CGRP neuropeptide was observed to be immunonegative in the testes of the young and subadult bovine, while in the adult, solitary CGRP fibers were demonstrated in the connective tissues of spermatic cord, cranial *mediastinum*, tunica albuginea, and mesorchium. Positive SP solitary fibers were seen in the ligamentum bridge situated between the epididymis and testis at the caudal testicular pole in the younger calf. However, SP immunoreactivity was negative in other age groups.

### Camel Testis and Epididymis: Localization and Distribution of Neuropeptides

Saleh et al. (36) investigated some selected neuropeptide (NPY, VIP, CGRP, and SP) distribution in the testis of camel. Neuropeptide Y was reported to be the predominant peptidergic transmitter in the testicular nerves of the camel, and it was colocalized with noradrenaline in the same axons. The VIP-containing fibers entirely accessed the camel testis as components of the caudal nervous contribution *via* the ligamentous bridge connecting the testis and epididymal tail and were restricted to the caudal pole of the testis. Interestingly, the CGRP-positive nerve fibers were not regularly demonstrated in the camel testis; however, Saleh et al. (36) reported the localization of this neuropeptide in stroma of spermatic cord, *tunica albuginea, septula, and mediastinum* testis as well as in the arterial walls containing varicose nerve terminals where they appeared to be the most vital sensory pathway of this organ. Substance P-positive axons constituted the fewest and appeared as solitary positive fibers in all parts of the testis, particularly at the cranial and caudal poles.

More recently, Liguori et al. (37) studied the distribution pattern of some neuropeptides (NPY, CGRP, and SP) in the epididymis of the alpaca camelid (Vicugna pacos) with the study presenting a strong evidence of colocalization of tyrosine hydroxylase and neuropeptide Y in most positive nerve fiber bundles demonstrable in the epididymal tubular interstitial connective tissue, in the periductal coats as positive varicose fibers, in the perivascular regions within interstitium, and specifically on the epithelial cells of the caput segment. There was a marked increase in NPY-positive nerve fibers from caput to cauda segments. With regard to CGRP and SP, the authors observed an irregular distribution of these nerve fiber bundles in the epididymal interstitial connective tissue.

### Cervine (Deer) Testis and Associated Structures

The report of Wrobel and Schenk (29) on the impact of seasons and age on testicular innervation in the cervine provided an insight into the distribution of neuropeptides (NPY, CGRP, VIP, and SP) and their colocalization with dopaminergic neurotransmitters (dopamine beta hydroxylase (DBH) in the nervous structures of the testes and associated structures (pampiniform plexus, mesorchium, epididymis, and ductus deferens) in red, roe, and fallow deers of different age groups. Generally, most vascular nerve fibers were postjunctional sympathetic axons with strong immunopositivity for DBH coexpressed with NPY in regions like the pampiniform plexus and testicular parenchymal tissue. Except for the spermatogenetically active fallow deer bearing seminiferous tubules surrounded by CGRP-positive nerve fibers, the interior of roe and red deer testes are devoid of CGRP nerve fibers. In all the species of deer studied, testicular *mediastinum*, vas deferens stroma, and *mesorchium* all had CGRP-positive axons. Considering the distribution of CGRP in deer testes, Wrobel and Schenk (29) suggested that the viscerosensory quality in the testicular intrinsic innervation was very likely to be mediated by the CGRP (calcitonin gene-related peptide)-positive fibers that run independently from the testicular vessels and end in the connective tissue of spermatic cord and tunica albuginea. Immunopositive VIP-nerve axons were restricted to the tunical and septular arteries of the testes in all the species of deer investigated. In terms of abundance, the gonad of red deer contained more immunopositive nerve axons than their counterparts, fallow and roe deer (29). It is important to mention that the authors also documented that in all the species of deer studied, there was no SP-positive axon in the cranial sympathetic nerve supply to the testis. Although rare, SP-positive axon occurs in the connective tissue of the *tunica albuginea* of the fallow deer or in the pampiniform plexus of the red deer.

### Donkey Testis and Associated Structures

The neuropeptide distribution in the donkey testes and associated structures (pampiniform plexus, spermatic cord, and epididymis) was profiled by Wrobel and Moustafa (25). Neuropeptide Y was colocalized with DBH and TH in the vast majority of postganglionic sympathetic axons of the testicular artery in the spermatic cord and along the epididymal side of the testis. There was no immunoreactivity to VIP in the interior of the testes of the donkey. The immunopositive CGRP fibers were observed in the wall of the testicular blood vessels, within the spermatic cord stromal connective tissue, and in the *tunica albuginea* and septula testis. The SP was rarely demonstrated in the *tunica albuginea*, in the vascular cone of the spermatic cord, and in the form of solitary fibers within vascular mural elements. Contrary to this observation, Arrighi and Domeneghini (38) had previously reported an exclusive localization of positive NPY nerve fibers in the perivascular portion of the testis of the donkey and negative immunoreactivity to peptides like CGRP, SP, Met-1, and Leu-1 enkephalin, and bombesin (BOMB)/GRP in the testis.

The NPY nerve fibers in the donkey epididymis were localized in the peritubular connective tissue, in the muscular tunic of the *ductus deferens* and the *lamina propri*a of the tunica mucosa as well as in muscular coat and axis of *ampulla ductus deferentis* (38). Also, substance P in the donkey epididymis was localized as long varicose nerve fibers within the interstitial spaces of the epididymal duct, as scattered single SP nerve fibers in the peripheral connective tissue of the duct and with a consistent display of decreased SP immunoreactivity from the head to the tail of epididymis (38). The authors observed that CGRP-positive nerve fibers in the epididymis of the donkey were detected as either small nerve bundles in the epididymal interstitium or as long varicose fibers in the muscular tunics of the epididymal duct and seemed to progressively decrease from caput to caudal epididymal segments (38). In the ductus deferens of the donkey, VIP immunoreactivity was observed to be limited to sparse nerve fibers distributed in the muscular coat.

### Horse Testis and Epididymis

In the horse testis, with the exception of the positive NPY nerve fibers that were exclusively restricted to perivascular location in the testis of the horse, immunoreactivity to neuropeptides like CGRP, SP, Met-1 and Leu-1 enkephalins, and bombesin/GRP neuropeptides were observed to be negative (38). Arrighi and Domeneghini (38) further reported a similar localization pattern of the donkey's epididymal NPY-positive nerve fibers in the horse, but with increased immunoreactivity. Also, the authors revealed that substance P distribution in the horse epididymis was equally similar to the pattern observed in the donkey. The CGRP-positive nerve fibers amount in the epididymis of horse were observed to decrease from the head to the tail of the epididymis and were detected either as aggregate of tiny nerve bundles in the interstitium of the epididymis, or as long varicose fibers in the muscular coat of epididymal duct.

### Buffalo Testis

The expression of neuropeptides in the testes of the buffalo was documented by de Girolamo et al. (39). Nerve bundles and solitary varicose neuropeptide Y fibers were intensely demonstrated in the *tunica albuginea* more particularly at the lateral and medial sides as well as the free border of the testis. The authors also observed NPY-positive immunoreactivity at the cranial and caudal testicular poles and surrounding blood vessels. Substance P immunoreactive fibers were sporadically localized at less intensity and density in the *tunica albuginea* and in the perivascular stroma of the testis when compared with NPY immunoreactivity. Other neuropeptides, such as GAL, VIP, CGRP, CCK, L-ENK, and M-ENK, were all immunonegative in the buffalo testis. However, there is no literature yet on the neuropeptide profiles of the buffalo extratesticular structures.

### Dog Testis and Associated Structures

Tamura et al. (40) investigated neuropeptide distribution in the testis and associated structures of dog using double-staining immunofluorescence study with confocal and fluorescence microscopic techniques. The study revealed that CGRP and SP were found to coexist in the nerve fiber bundles within spermatic cord and testicular visceral vaginal tunic as well as in the single fiber running along the surface of the testis. Interestingly, the authors demonstrated that vast majority of SP-positive fibers coexisted with CGRP; however, there were instances of CGRP-positive fibers without immunoreactivity to SP neuropeptide. In addition, Tamura et al. (40) employed immunogold silver staining for CGRP to localize CGRP-positive nerve bundles, single nerve fibers, and nerve terminals. The latter were specifically demonstrated in the tunica albuginea at about <5 μm from the surface.

### Cat Testis

The works of Larsson (41) and Alm et al. (42) were among the early sets of reports on neuropeptide distribution in the genital tract of the cat. The authors independently reported VIP immunoreactivity in the testis and ductus deferens of cats. Immunopositive VIP fibers were demonstrated in the following parts of the genital tract, testicular capsule, interstitium between efferent duct, *lamina propria* of the mucosa, and the smooth muscle coats of the *ductus deferens*.

The neuropeptide distribution (NPY, CPON, VIP, and SP) in the testes and associated structures (testicular pedicle, cranial spermatic, and testicular nerve fibers) in the cat was equally investigated by Suburo et al. (43). The study showed that numerous neuropeptide Y and C-terminal flanking peptides of NPY nerve fibers were localized in the testicular pedicle (CrSN bundles, arterial, and veinous walls) together with moderate population of NPY-positive fibers in the CdSN bundles. However, a varying range (scarce to numerous) of NPY/CPON-positive nerve fibers were localized in the testicular capsule, blood vessels, and interstitium. Regarding VIP, there exist conflicting reports on its localization. Suburo et al. (43) reported the detection of a few positive fibers in the CrSN, arterial and veinous walls in the testicular pedicle, and numerous VIP-positive nerve fibers in the CdSN, testicular capsule, blood vessels, and interstitium of cat testis. On the contrary, Wrobel and Giirtler (44) reported negative VIP immunoreactivity in their study. The number of calcitonin gene-related peptide-positive fibers seemed to be distinctively demonstrated in the testis with preferred locations being between the testicular pedicle, ISN bundles, interstitial Leydig cells, perivascular regions, the *tunica albuginea* stroma of the capsule, *mediastinum*, and testicular septa (43, 44). On substance P, immunoreactivity to SP nerve fibers was rarely localized in the *tunica albuginea* of the testicular capsule, *septula testis*, mediastinum, *rete testis*, interstitial Leydig cells, testicular lobules periphery, CdSN bundles, and intratesticular blood vessels (43, 44). It is also important to mention that Suburo et al. (43) reported the detection of very few to moderate Galanin-positive fibers in the testicular pedicle, ISN bundles, testicular capsule, intratesticular blood vessels, and testicular interstitium.

### Pig Testis and Its Investments

Age-related changes in the distribution of nerves access to the testis and its investment in pigs using pan-neuronal markers and neuropeptides have been described by Wrobel and Brandl (24). It was shown that the testes of piglets <7 days old have innervations restricted to the septal and mediastinal regions, while those between 3 and 5 weeks old bear the most intense and steady innervation, which reaches the gonad through three different routes: the funicular, caudal, and mesorchial innervations. Their testicular nerve fibers supply the vascular structures of the spermatic cord, the tunica albuginea, the septula testis, and the mediastinum. In the 7- to 10-week-old pigs, varying degrees of testicular innervation withdrawal were conspicuous, and in the adult boar testes, the funicular nerve supply to the testicular artery and pampiniform plexus were retained but with no evidence of intrinsic nerves. With regard to neuropeptide distribution across different age groups, NPY constituted the predominant (about 75%) neuropeptides in pig testicular nerves. The NPY-positive nerve fibers were demonstrated in the *tunica albuginea*, seminiferous tubular interstitium, *mediastinum testis*, and *septula testis*. The VIP nerves were demonstrated in the funicular and supratesticular vascular stroma and in the fibers accompanying the epididymal arteries to the epididymal head. The CGRP formed about 50% of nerve fibers in the subadult and was localized in the intervascular stroma, testicular vascular organ, and *tunica albuginea*. However, CGRP fibers were less frequently demonstrated in the *septula testis* and the *mediastinum*. In case of the adult boars, CGRP nerve axons were markedly reduced. A small population of about 10% nerve bundles were immunopositive for SP in all age categories.

A previous study by Kaleczyc et al. (45) revealed that non-noradrenergic putative cholinergic, noradrenergic, and sensory nerve terminals were the three main populations of nerve fibers supplying the accessory genital glands of pigs. A more recent study showed that VIP and NPY, and, to a lesser degree, SOM are associated with the cholinergic nerve fibers supplying the accessory sex glands (46).

### Rabbit Testes and Associated Structures

Sienkiewicz et al. (22) reported neuropeptide distribution and colocalization in the testes, epididymis, and vas deferens of rabbits. Neuropeptide Y was either demonstrated in single sparse form or majorly colocalized with DBH in most nerve fibers supplying the highlighted structures. In terms of immunoreactivity, low positive immunoreactivity was found in the *tunica albuginea*, vascular elements, and *mediastinum* of the testis, head, body, and tail segments of epididymis as well as the furnicular and abdominal parts of the ductus deferens. There was no immunoreactivity within the spermatogenic elements of the seminiferous tubule. The immunoreactivity was demonstrated only in the *tunica albuginea* and vascular stroma of the testis, the three segments of the epididymis, and ductus deferens (both furnicular and abdominal). Moderately positive CGRP immunoreactivity was observed in the *tunica albuginea* and vascular stroma of the testis, while marked immunoreactivity was restricted to the various epididymal ductus deferens segments. In the case of SP, a similar immunoreactivity density observed for CGRP was displayed in both testis and epididymis.

### Rats' Testis and Associated Structures

Properzi et al. (47) investigated postnatal (prepubertal to adulthood) changes in the distribution and relative density of some neuropeptides (NPY, VIP, and CGRP) in the male genital tract (testis, epididymis, ductus deferens, seminal vesicles, prostate, and penis) of the Wistar rat. The authors reported negative immunoreactivity to all the neuropeptides in both the testicular parenchyma and caput epididymis. On age-related differences, the authors observed only very scattered CGRP-containing nerves in the muscular coat, tubular interstitium, and perivascular parts of both caudal epididymis and ductus deferens) of 8-day-old rats. Contrastingly, numerous immunopositive VIP, CGRP, and NPY peptide nerve fibers were demonstrated in the cauda epididymis and ductus deferens of 20-day-old rats. Interestingly, the population of the immunopositive peptidergic nerves increased in 35-day-old rats, while in the more adult rats, there were no visible changes. The postnatal developmental study conducted by these authors on the ontogeny of peptidergic nerves seemed to coincide with the differentiation of the epididymis and ductus deferens, thus, signifying that the potential neuropeptides released from these peptidergic nerves could have participated *via* probable reciprocal influences with target cells to bring about the morphofunctional postnatal development of the genital system in male rats.

Also, the profiles of the two major neuropeptides (NPY and VIP) were investigated in the testis of rats by Rauchenwald et al. (11). The NPY was seen throughout the inner surface of the testicular capsule with fibers penetrating the *tunica albuginea*. In addition, the NPY-positive fiber varicosities were sparsely distributed throughout the testicular interstitial space and appeared to be associated with microvessels and along the seminiferous tubules. The authors did not detect immunoreactivity to VIP in the rat testis. This observation is partially in tandem with negative immunoreactivity to NPY, VIP, and CGRP neuropeptides as observed by Properzi et al. (47) in both the testicular parenchyma and caput epididymis of Wistar rats during their postnatal life. More recently, in the quest to further understand the distribution of neuropeptides in the testes of rats, Allen et al. (48) immunohistochemically localized NPY subreceptor in the smooth muscle layers of the testicular vessels that are situated between seminiferous tubules where they were suggested to exert local vascular impairment on gonadotropin delivery to, and/or blunted testosterone secretion from, Leydig cells. There was no direct immunoreactivity in the Leydig cell of the rat studied.

The innervation of the *ductuli efferentes* and seven zones of the guinea pig epididymis was investigated by Greenberg et al. (49) using three approaches: immunohistochemistry, histochemistry, and electron microscopy. With the use of neuropeptide markers (SP, VIP, and DBH), the authors localized immunopositive SP and VIP nerve fibers consistently in the interstitium, muscular coat, and perivascular areas of the efferent ducts and the various zones of the epididymis. Similarly, immunopositive DBH nerve fibers were restricted to the interstitial and perivascular regions of all the zones of the epididymis. The histochemistry results corroborated their immunohistochemistry findings as a substantial amount of acetylcholinesterase-containing fibers were observed in the interstitial, perivascular, and periductal smooth muscles of the *ductuli efferentes* and a few zones (V, VI, and VII) of the epididymis. Ultrastructural features of cholinergic (agranular vesicles), adrenergic (small granular vesicles), and peptidergic (large granular vesicles) innervations were seen in the interstitial connective tissue of both the efferent duct and the various zones of the epididymis. The localization of these selected peptidergic nerve fibers in some specific regions of the efferent duct and epididymis has been suggested to portend ranging physiological roles including SP mediation of smooth muscle constriction in the epididymal parenchyma and its perivascular structures, and VIP initiation of smooth muscle dilatation at the caudal aspects of the epididymis, the seat of spermatozoa storage.

Functionally, the ultrastructural features of nerve chemical coding noted by the authors implied a costorage of adrenergic, cholinergic, and peptidergic transmitters in the epididymis of guinea pig.

### Rhesus Monkey Testis

Frungieri et al. (26) documented NPY profiles in different age categories (infantile, juvenile, and adult) of Rhesus monkeys. The study showed that nerve fiber immunoreactivity for NPY were localized along the seminiferous tubular wall, in the seminiferous tubular interstices, and in vascular stroma of the testis of all age groups.

## Neuropeptidergic Outline of the Male Accessory Glands

The male sex accessory organs, just like the main sex organs, are supplied by nerves containing not only the classical neurotransmitters, such as acetylcholine and noradrenaline, but also a vast array of peptidergic transmitter candidates (50). Some of the most widely distributed neuropeptides documented to date include neuropeptide Y, vasoactive intestinal peptides, calcitonin-related gene peptides, substance P, Met-1 and Leu-1 enkephalin, galanin, and somatostatin (35, 46, 51). Below is an overview of the mammalian male accessory sex glands neuropeptide profiles.

### Seminal Vesicular Neuropeptide Distribution in Man, Rat, and Guinea Pig

A considerable quantity of NPY-immunoreactive fibers has been localized in male accessory sex glands receiving dense sympathetic nerve supply (15). NPY nerve fibers have been localized in the smooth muscle layer, subepithelial connective tissue, and perivascular tissues of the seminal vesicular gland of man and rodents, and seem to be the dominant peptidergic nerve fibers (34, 35, 47, 52–58).

Positive CGRP immunoreactive single and thick bundles of nerve axons have been localized in the perimuscular coat, sub-epithelial connective tissue, and in the perivascular spaces of the seminal vesicular gland in the guinea pig, man, and hamster rat (35, 51, 53, 59). Substance P peptide has been colocalized in the afferent nerve fiber supply to seminal vesicle with CGRP-positive fiber population outnumbering the SP nerve fibers (60).

Positive immunoreactivity to PHI has also been documented in man (35), rat, and guinea pig seminal vesicles (53) with man vesicular gland displaying abundant PHI nerve fibers compared with the less numerous fibers demonstrated in guinea pig and rat seminal vesicles (53). The authors also suggested possible colocalization of VIP and PHI.

The VIP nerve fibers were majorly localized on the basal part of the seminal vesicular gland and within the muscular coat as well as in vascular elements of the gland in smaller amounts (34, 35, 53, 61–65). With the use of tissue autoradiography and binding assays, VIP binding site was exclusively localized in the seminal vesicle secretory epithelium of rats and hamster rats, and to some extent, in the muscular coat and in the vascular wall of medium vessels in hamster rats (50).

Positive immunoreactive SP, enkephalins, and gastrin-releasing peptide nerve fibers have been demonstrated in varied quantities in the smooth muscle and mucosa of the seminal vesicles of man, rat, and guinea pig (34, 35, 53, 61, 63–65). Of all these neuropeptides, gastrin-releasing peptide had the least immunoreactivity in the smooth muscle layer of the gland, while enkephalins remained the most controversially expressed peptide in the seminal vesicle (15, 35, 61). Also, Tainio (35) reported that the seminal vesicle of man was immunonegative for GAL nerve fibers.

On the possibility of age-related differences in the neuropeptide localization and expression in accessory sex glands, Properzi et al. (47) reported differences in the postnatal localization of three peptidergic (VIP, CGRP, and NPY) nerve fibers in the seminal vesicle and the prostate gland of the Wistar rat. The authors observed that NPY nerve fibers remained the dominating fibers of the three peptidergic nerve fibers and was localized in both the glandular interstitium and muscular coat around the prostate and seminal vesicle. Meanwhile, immunopositive VIP and CGRP nerve fibers were demonstrated in the subepithelial and perimuscular coat of the glands, respectively. Properzi et al. (47) also noted that the three neuropeptides mentioned above were immunonegative in the accessory glands of 8-day-old rats and numerously demonstrated in the glands of 20-day-old rats. Similarly, the immunoreactivities for the selected peptides were markedly increased in 38-day-old and above age categories.

### Neuropeptide Distribution in the Seminal Vesicle of Pig

Klimczuk et al. (46) investigated the occurrence and colocalization patterns of VAChT, dopamine β-hydroxylase (DβH) and some selected neuropeptides [VIP, NPY, and somatostatin (SOM) in the accessory gland (seminal vesicle, prostate, and bulbourethral)] of male pigs using both the single and double immunohistochemical labeling. The study findings revealed that the aforementioned biologically active substances in nerve axons innervating particular glands were demonstrated beneath the epithelium, in the interstitium, and within vascular structures of the glands, where they all shared similar coexistence though with varying density of cholinergic innervation between organs. For instance, in terms of cholinergic innervation density, the seminal vesicle and prostatic body seemed to be well-developed relative to the disseminated portion of prostate and bulbourethral gland. Of note, a key finding from the study of Klimczuk et al. (46) was that the vast majority of the cholinergic nerve fibers located around the vascular structure to the glands contained VIP and NPY.

### Seminal Vesicular Neuropeptide Distribution in the Donkey and Horse

Regarding neuropeptide distribution in the seminal vesicle in the donkey and horse, Arrighi and Domeneghini (38) documented a comprehensive comparative distribution of diverse neuropeptides (SP, CGRP, Met 1 and Leu 1 enkephalins, and bombesin). From the study, immunoreactive SP nerve fibers were observed only in the seminal vesicle of the donkey and were confined to the interstitium, muscular coats, and periglandular lobules. The CGRP fibers were detected in small quantities in the interstitium of vesicular glands of the horse. Concerning VIP immunoreactivity in both horse and donkey, immunopositive nerve fibers were markedly localized in the interstitial spaces surrounding muscle coats and subepithelia part of the bulbourethral gland and rarely or scantly demonstrated in the prostate gland interstitium.

Met-1 and Leu-1 enkephalin-positive nerve fibers were demonstrated in the muscular layer of ductus deferens and its ampulla as well as subepithelial connective tissue of ductus deferens in the horse and donkey, although the donkey had an exclusive demonstration of positive Met-1 and Leu-1 enkephalin nerve fibers in the external muscular layer of caudal epididymis, ductus deferens, ampullary, and vesicular glands. For bombesin/GRP neuropeptide, positive bombesin nerve fibers were exclusively detected in the muscular tunic of the ductus deferens in the horse but restricted to the perivascular regions of ductus deferens in the donkey. Somatostatin peptide was immunonegative to the nervous, connective, muscular and epithelial components in the testis, epididymis, ductus deferens, and accessory glands in the horse and donkey (38).

### Prostate Glands of Man, Guinea Pig, Mouse, and Rat

The report of Higgins and Gosling (66) on using acetylcholinesterase (AChE) activity and neuropeptide immunoreactivity to unravel prostate gland innervation in adult men showed that the distribution and density of AChE-positive nerves associated with smooth muscle in either the peripheral or central parts of the prostate were indistinguishable. Also, it was noted that the vast majority of the peripherally and centrally located acini had conspicuous subepithelial plexus of autonomic nerves. With respect to neuropeptides, the authors observed that immunopositive VIP nerve fibers were localized along the epithelial lining of acini in both the central and peripheral portions of the gland. However, NPY-positive nerve fibers were exclusively restricted to the smooth muscle trabeculae of the prostate gland. It was also on record that neuropeptide Y was colocalized in both the adrenergic and cholinergic nerves in the pelvic ganglia (16). The prostate gland stroma in man, guinea pigs, and rats were recognized for the demonstration of dense NPY nerve fibers (67–70).

Although there are conflicting reports of dual possible demonstration of NPY immunopositive nerve fibers in the human prostate (35, 66). The first category of report localized NPY nerve fibers in the prostatic stroma, and the second demonstrated it in association with prostatic acini epithelium (67, 71). Similarly, there are pieces of evidence of conflicting reports with regard to the expression of NPY receptors in the prostates of humans and rats (72, 73). On the variation in the localization of NPY, Pennefather et al. (68) reported the distribution of neuropeptide Y, thyroxine hydroxylase, and acetylcholinesterase in the guinea pigs' prostate gland section. The NPY immunopositive fibers were demonstrated in both the glandular stroma and epithelium. Similarly, cholinesterase-reactive fibers were observed in the stroma and epithelium of the prostate gland, while immunopositivity for TH was restricted to only the smooth muscle tissue around the gland.

The prostate gland in most animals is rich in VIP nerve fibers and constitutes the dominant colocalized peptides in the cholinergic nerve supplying the glandular epithelium of the prostate gland (16, 62, 69). Apart from nerve axons demonstration, VIP receptors have equally been expressed in the prostate gland (74). On the demonstration of calcitonin gene-related peptide (CGRP) nerve fibers, positive fibers were localized in the prostate gland stroma and epithelium as well as in the neuroendocrine cells of the prostate of several mammalian species (70, 75–78).

Neurokinin and substance P have been sparsely localized in nerve fibers innervating the smooth muscle tunic of man, sheep, dog, guinea pig, rat, and mouse prostate glands (67, 77, 79–81). For galanin neuropeptide localization, sporadic GAL-positive nerve fibers were demonstrated in the prostate of hamster rats (82), while in the prostate of the Wistar rat, no GAL nerve fiber was demonstrated (83).

### Neuropeptide Distribution in the Prostate Gland of Monkeys

Yokoyama et al. (84) investigated nerve fiber distribution in the caudal lobe in the prostate of monkey, *Macacus fuscatus*, using both histochemical [acetylcholinesterase (AChE) reactivity] and immunohistochemical (TH and NPY immunoreactivities) approaches. The authors observed abundant positive TH-like immunoreactive nerve fibers in the interstitium and infrequent distribution in the paraurethral region. Also, NPY-like immunopositive nerve fibers were localized in the interstitium and periprostatic acini on a less abundant basis compared with the TH counterpart. However, AChE-positive nerve fibers were densely demonstrated around the acini compared with the interstitium. The authors also noted that both NPY-like and AChE-positive nerve fibers seemed to be localized in close association with the acini epithelium.

### Neuropeptide Distribution in the Prostate Glands in Cats and Dogs

The available report on peptidergic nerve fiber distribution was conducted by Smith et al. (74) with the use of indirect immunofluorescence. The author demonstrated an irregular distribution of thin, beaded VIP-immunoreactive nerve fibers parallel to the smooth muscle coats and interstitial tissues ramifying the prostatic acini. Also, immunopositive VIP fibers were infrequently localized in the glandular epithelium and perivascular aspect of the gland. For the cat prostate gland, Larsson (41) and Alm et al. (42) demonstrated immunopositive VIP nerve fibers in the prostate gland capsule and the intervening stroma between the acini.

### Neuropeptide Distribution in the Prostate Glands of Donkeys, Horses, and Rams

Arrighi and Domeneghini (38) observed that the prostate glands of both horse and donkey especially the interstitium, muscular coats, and periglandular lobules were remarkably immunopositive for NPY nerve fibers. Also, the authors localized sparse SP-positive nerve fibers in the glandular fold axis, the interstitium that closely apposed the smooth muscle layer, the muscle coat of small arteries, and perivascular regions of the gland.

Arciszewski (81) employed double immunohistochemistry to investigate localization and distribution patterns of peptidergic (CGRP, SP, and GAL) fibers in seminal vesicles and prostate of the ram. Localization-wise, CGRP and SP immunoreactive (IR) nerve fibers were demonstrated in the mucosa and smooth muscular tunics of the seminal vesicles and prostate with CGRP-positive fibers outnumbering the SP fibers, while immunopositive GAL nerve terminals were sparsely localized relative to other peptides. Arciszewski (81) also observed strong colocalization between CGRP-positive fibers and SP as well as between SP and GAL fibers.

### Commentary on the Interspecies Variations in the Neuropeptide Distribution of the Male Genital Tract

We provided a concise overview of the neuropeptide profiles in the male genitalia of various mammalian species in Table 1 (with the corresponding references), and as a follow-up to this, a concise comparative note on the interspecies variabilities of the neuropeptides is stated below.

**Table 1:**
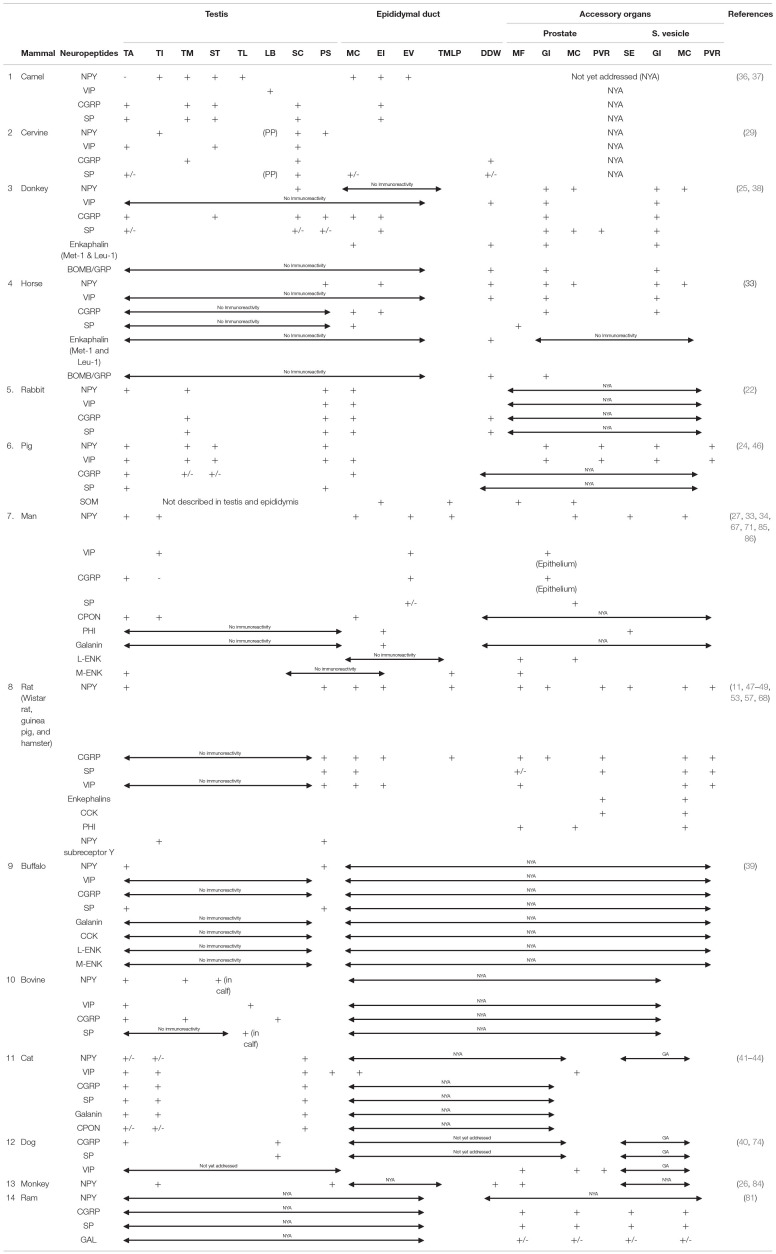
Summary of the Neuropeptide profiles of the male genitalia in various mammalian species.

*+, Abundant nerve fiber presence; +/-, rare/scanty/sporadic/ocassional nerve fiber presence; TA, tunica albuginea; TI, tunica interstitium; TM, testicular mediastinum; ST, septular testis; TL, testicular lobules; LB, ligamentum bridge; SC, spermatic cord; PS, perivascular stroma; MC, muscular coat; EI, epididymal interstitium; EV, epididymal vessels; TMLP, tunica mucosa-lamina propria of epididymis; DDW, ductus deferens wall; MF, mucosal fold; GI, glandular interstitium of prostate or seminal vesicle; MC, muscular coat or tunic around glands; PV, perivascular region; PE, prostate epithelium; SE, seminal vesicle epithelium; GA, gland absent; PP, pampiniform plexus; NPY, neuropeptide Y; VIP, vasoactive intestinal peptide; CGRP, calcitonin gene-related peptide; SP, substance P; L-ENK, leu-enkephalins; M-ENK, met-enkephalins; CPON, C-terminal flanking peptide of NPY; PHI, peptide histidine isoleucine; BOMB, bombesin; CCK, cholecystokinin*.

*N:B: neuropeptides distribution in the bulbourethral gland in these species are as follows:*

*Pig: Similar to the pattern described for prostate and seminal vesicle*.

*Horse: NPY: + (PV), VIP: (PV), M-LENK: (epithelia, PV), L-LENK (epithelia), BOMB/GRP (PV)*.

*Donkey: NPY (PV)*.

### Testes and Associated Structure Peptidergic Interspecies Variations

#### Neuropeptide Y

Comparatively, the neuropeptide Y has broad immunopositive areas in the testis of man, monkey, rabbit, cat, pig, and camel. The involved parts of the testis in these species include the testicular capsule, peritubular myoid cells, seminiferous tubular interstitium, *mediastinum testis*, and vascular stroma within and around the testis. With respect to testicular NPY distribution in the buffalo, rat, and cervine, NPY was demonstrated in the testicular capsule only and/or in the vascular stroma as well as seminiferous tubular interstitium. The mammalian species with the most restricted distribution area (perivascular tissue) of testicular NPY are the donkey and horse. Surprisingly, there is no report yet on the NPY distribution in the dog.

### Vasoactive Intestinal Peptide

On the distribution of VIP nerve fibers, the widest immunoreactive testicular regions (*tunica albuginea*, testicular mediastinum, interstitium, and perivascular stroma) were observed in the rabbit, pig, bovine, and cat. This was followed by the camel, cervine, and man with one or two regions (ligamentous bridge, spermatic cord/perivascular stroma, and interstitium) of VIP immunopositivity (Table 1). There are no existing reports of VIP immunoreactivity in the testes of the monkey, buffalo, horse, donkey, dog, and ram.

### Calcitonin Gene-Related Peptide

Concerning the distribution of CGRP in the testicular parenchyma and associated structures, the broadest distribution is found in the camel, donkey, rabbit, pig, bull, and cat. The specific areas in these animals include the *tunica albuginea, tunica mediastinum, septular testis*, spermatic cord, testicular interstitium, and perivascular stroma. The cervine, man, and dog possessed either one or two areas (*tunica albuginea*, testicular mediastinum, and spermatic cord) of immunopositivity. There was negative immunoreactivity in the testes of the horse, rat, and buffalo. However, in the monkey and ram, there is no available literature on the distribution of CGRP nerve fibers in their testes.

#### Substance P

The camel, donkey, and cat testes have the most enormous SP-positive nerve fiber distribution in the *tunica albuginea*, testicular mediastinum, *septular testis*, perivascular stroma, and spermatic cord. Unlike other mammals [cervine, rabbit, pig, buffalo, bovine (specifically male calf), and dog], immunopositive areas were restricted to one or two areas in the testes. Interestingly, there was negative SP immunoreactivity in the testes of the horse, man, and rat. Also, there is a dearth of literature on SP nerve fiber distribution in the testes of the ram.

#### Enkephalin

The reports available for interspecies variation on enkephalin distribution in animals under review are very scanty for meaningful comparison. Enkephalin-positive fibers in the testis were localized only in the *tunica albuginea* of man's testis, but immunonegative in the testes of the donkey, horse, and buffalo. There are no existing literatures on the testicular immunoreactivity in other animals. With the exception of positive immunolocalized enkephalin fibers in the muscle coats of epididymis of the donkey and *ductus deferens* wall of both the horse and donkey, there is scarcity of literature on its presence or distribution in other animals.

#### Galanin

Galanin-positive nerve fibers were found only in the cat testicular capsule, interstitium, and perivascular stroma. However, it was immunonegative in the testes of man and buffalo. There is little or no report on its immunoreactivity in other animals.

### Epididymal Peptidergic Interspecies Variabilities

#### Neuropeptide Y

Neuropeptide Y has been consistently immunolocalized in the epididymal interstices and perivascular structures in man. In the donkey and horse, it is exclusively restricted to the peritubular connective tissue. With the exception of NPY immunolocalization on the epididymal epithelium, the camel shared similar NPY distribution with man. There is still a wide literature gap in the demonstration of NPY in the epididymis of quite a lot of mammals including the bull, cervine (deer), buffalo, dog, cat, and monkey.

#### Vasoactive Intestinal Peptide

There is a dearth of information on the VIP immunoreactivity in the epididymis of the camel, cervine, donkey, horse, buffalo, dog, and ram. However, vast VIP-immunopositive regions (epididymal interstitium, vasculature, and muscular coats) were exclusively observed in the rat epididymis. The rabbit, pig, and man come next in the extent of distribution of VIP nerve fibers in the epididymis. These sets of animals were immunopositive for VIP in either the epididymal muscular coat or the interstitium. For ducutus deferens, the VIP-rich area is in the muscular coat of the donkey, horse, rat, bull, and cat. There are no existing reports on other animals in this regard.

#### Calcitonin Gene-Related Peptide

With the exception of the rat bearing three immunopositive CGRP areas in the epididymis (epididymal muscle coat, interstitium, and vessels), every other mammal (camel, donkey, horse, rabbit, man, and cat) with immunoreactivity had one or two areas positive for CGRP fibers. There are no available reports on the immunoreactivity of CGRP in the epididymis of the cervine, pig, buffalo, bovine, dog, monkey, and ram. The CGRP immunoreactivity in the ductus deferens is reported to be demonstrated in the muscular coat or wall of the cervine, rabbit, and rat, while there is still a huge literature gap in the CGRP fiber presence in the ductus deferens of other animals.

#### Substance P

The SP-positive nerve fibers had exclusive distribution in one or two regions (muscular coat and interstitium) of the epididymis in the camel, cervine, donkey, horse, rabbit, man, and rat. There is a dearth of literature on its distribution in the epididymis of other animals in this review. On species variation in the SP nerve fiber distribution, immunopositive fibers are restricted to the wall or muscle coats of the epididymis in the cervine, donkey, and rabbit. There are no reports on their immunoreactivities in other animals.

### Peptidergic Interspecies Variations of the Prostate Gland

#### Neuropeptide Y

Of all the prostates of animals in this review, the donkey, horse, pig, man, rat, and monkey had immunopositive NPY fibers. The NPY immunoreactivities in these animals were confined to one of these parts (muscle coat, perivascular stroma, glandular interstitium, and epithelium) of the prostate gland. However, there is a dearth of literature on NPY immunoreactivity in other animals.

#### Vasoactive Intestinal Peptide

The positive VIP immunoreactivity was demonstrated mainly in the muscle coat of the prostate gland of six mammalian species (donkey, horse, pig, man, rat, and monkey) out of the total animals under review. Although the VIP-positive fibers were additionally localized in the perivascular stroma of pig, there is no report on the VIP immunoreactivity in the prostate of other animals.

#### Calcitonin Gene-Related Peptide

The positive prostatic CGRP fibers were demonstrated in the donkey, horse, man, and rat in two specific locations: glandular interstitium and prostatic epithelium. The prostatic profile of CGRP is still scanty in other animal species.

#### Substance P

In all the animal species in the review, only five mammals (donkey, horse, man, rat, and ram) had positive prostatic SP immunoreactivity precisely in the glandular interstitium, muscle coat, and perivascular stroma. The prostatic SP immunoreactivity is still poorly investigated in other mammals.

#### Galanin

The only prostatic galanin immunopositive fibers in this review were found in the ram. However, the rest of the other mammalian species still lack documentation on the galanin neuropeptide.

#### Enkephalin

On the variation in the demonstration of enkephalin, the donkey is the only species with prostatic enkephalin (leu and met)-positive nerve fibers. Both horse and rat had negative enkephalin immunoreactivity. Generally, the information on prostatic enkephalin immunoreactivity is still lacking in other species.

### Peptidergic Interspecies Variations of the Seminal Vesicle

There is no seminal vesicular gland in carnivores (6). Therefore, the lack of information on neuropeptide distribution in cat and dog seminal vesicles is understandable.

#### Neuropeptide Y

The NPY-positive nerve fibers in seminal vesicular gland were demonstrated in four mammalian species (donkey, horse, pig, man, and rat) in diverse parts of the gland including the muscle coat, interstitium, perivascular stroma, and epithelium. However, the NPY distribution is still lacking in the remaining mammalian species under consideration.

#### Vasoactive Intestinal Peptide

The VIP-immunopositive fibers were variously localized in the muscle coat, interstitium, and perivascular stroma of the seminal vesicle in the horse, donkey, pig, and rat. There is a lack of documentation on VIP neuropeptide distribution in the seminal vesicle of other mammalian species.

#### Calcitonin Gene-Related Peptide

On the differences in the distribution of CGRP neuropeptide, markedly enormous CGRP-positive nerve fibers were observed in the rat and ram especially in the seminal vesicular epithelium, muscular coat, and perivascular stroma. The CGRP-nerve fibers in the donkey and horse were demonstrated only in the seminal vesicular gland interstitium. There is no information on seminal vesicular CGRP neuropeptide distribution in other mammalian species.

#### Substance P

The SP-positive immunoreactivity in the seminal vesicle, so far, was found to be widely distributed in the glandular interstitium, epithelium, and muscle coat of the rat. The donkey and ram had fewer areas of SP immunopositivity compared with the rat. The SP immunoreactivity was negative in the seminal vesicle of the horse. There is a lack of information on SP immunoreactivity in the selected mammalian species.

#### Bombesin and Galanin

With the exception of immunopositive bombesin nerve fibers in the interstitium of seminal vesicular gland, there is no information on its distribution in other mammalian species. Similarly, galanin neuropeptide was demonstrated in the epithelium and muscle coat of seminal vesicle of the rat. There is no data on galanin immunoreactivity in other animal species.

#### Functional Implication of Neuropeptides in the Testis and Epididymis

The prevailing functional assumptions for the frequent colocalization of catecholaminergic and peptidergic fibers in the testicular parenchyma have been linked to modulation of Leydig cell function since most Leydig cells display β adrenergic receptors (87). The general functional relevance of neuropeptide Y in the testicular physiology has been associated with the activation of the NPY Yl receptor in testicular tissue, which mediates vasoconstriction in collaboration with noradrenaline or counteracts VIP-induced vasodilation (72, 88, 89). The VIP is a recognized potent vasodilator found in non-adrenergic nerves (90, 91). The CGRP is a neuropeptide that is released at capsaicin-sensitive sensory nerve endings within the genital tract with crucial physiological roles in touch and pain sensations, and capable of involving its somatic innervation by the genitofemoral, pudendal, inguinal, and cutaneous femoris posterior nerves (18, 92, 93). Functionally, the assumption made on testicular physiology could be extended to the epididymis regarding NPY, which appeared to be a potent modulator of adrenergic neurotransmission required in sperm ejaculation and transport (53, 94) as well as regulation of blood flow (33). The possibility of the involvement of SP and CGRP in regulation of pain sensation in the genital organs has also been established (95).

#### Functional Implications of Neuropeptide Localization in the Mammalian Accessory Sex Glands

The NPY, being the predominant neuropeptide in the muscle layer of the seminal vesicles, has been presumed to play a key functional role in the regulation of the glands' smooth muscle tone, although several conflicting reports of other neurotransmitters responsible for the motor transmission in the seminal vesicles have been established (55, 58, 96–98). In addition to the presumptive function, NPY has been suggested to participate in most of the epithelial secretory functional regulation in the male accessory sex glands. This was predicated on the presence of either NPY nerve axons around the basal epithelium of the seminal vesicular gland or the presence of NPY receptors in epithelial cells in ventral prostate of rats (15, 99). In addition to these functional implications, the localization of NPY-like nerve fibers around the prostatic acini in the prostate of monkeys has been implicated in the regulation of the cellular secretion by acting on the cholinergic nerves as well as by having a direct effect on the acinar secretory cells (84). Regarding the morphofunctional assumptions on NPY role, Watts and Cohen (100) contrarily observed that NPY does not elicit any effect on the glandular contractility.

The immunolocalization of CGRP within the prostatic epithelial and subepithelial regions of most mammals especially guinea pigs has been associated with the regulation of prostatic secretion (101). Although numerous workers are of the opinion that NPY does not seem to regulate or facilitate prostatic contraction but rather participate in prostate growth (73, 100, 102, 103), however, exogenously administered high concentration of NPY has been reported to inhibit nerve-mediated concentrations and relax noradrenalin precontracted prostate preparations (78).

The physiological role of CGRP in seminal vesicles has been linked to its inhibitory modulation *via* its smooth muscle relaxant property on the autonomic regulation of contractility in the seminal vesicle (51). Also, CGRP nerve fibers in close proximity to seminal vesicle epithelium have been suggested to participate in influencing its secretory processes (104, 105). The functional relevance of CGRP in the prostate gland has been inferred from its relaxant effect on phenylephrine-mediated contractions or its inhibitory role in nerve-mediated contractions in the rat prostate (100). The demonstration of abundant CGRP- and SP-immunopositive nerve fibers in the seminal vesicle and prostate glands of ram has been implicated in active participation in the sensory transmission and regulation of smooth muscle contractility (81).

Functionwise, VIP has been found to affect secretory activity and contractility of the seminal vesicles in hamster rats (50). Due to the established vasodilating property of VIP together with localization of VIP-binding sites within smaller arteries and arterioles in hamster rat vesicular gland, it is presumed that these facts could contribute to seminal vesicular gland secretion (106). Besides the documented active participation of VIP in penile erection (107), it is also assumed to play an active role in muscle relaxation, blood flow, and secretory activity *via* probable antagonism of NPY action on the reproductive organs (38). The antagonistic effect of NPY has been observed in rats, where it manifested in the form of inhibition of VIP-induced cAMP accumulation in the rat prostate, although the participating structural aspects of the gland was more of epithelium than stroma (99, 108).

By virtue of the distribution of VIP receptors in the prostate gland, it is presumed that it has little or no participation in glandular contraction, rather secretory and prostatic growth functions seemed appropriate (74). The subepithelial presence of VIP nerve fibers in the accessory glands of rats during postnatal days 20 and 38 days and above has been presumed to be functionally important in influencing the epithelial function of the accessory glands (47). The immunolocalization of the NPY and CGRP in the muscular and perimuscular coats of the glands during the specified postnatal days (20 and 38 days and above) seemed to correlate well with the sequential morphological development of these organs.

Leu and Met enkephalins are opioid peptides that are usually localized in both endocrine cells and autonomic innervation. The functional relevance of these peptides, when found in the male genital tract of most mammals, has been associated with the regulation of smooth muscle activity of glandular organs, such as seminal vesicles, to release and transport seminal fluid (38). It has equally been assumed that adrenergic transmission could be maintained by substance P and at the same time inhibited by enkephalins within the rat vas deferens to modulate the process of ejaculation (109). It is also on record that neurokinin A and substance P could potentiate smooth muscle contraction occasioned by nerve stimulation in the guinea pig prostate gland (80), although there were reports on the absence of direct contractile effects of substance P on the prostate gland in pigs and rats (100, 110).

The immunolocalization of bombesin/GRP nerve fibers in the muscular coat and perivascular tissue of the ductus deferens and seminal vesicle in most mammals has been suggested to be involved in the induction of motor effects in the smooth musculature (65).

## Concluding Remarks

This review has laid to bare that the vast majority of neuropeptides present in the autonomic nerve supply to the different main and accessory genital organs in 14 species of mammals probably coexist with other peptides or with various neurotransmitters (tyrosine hydroxylase, dopamine beta hydroxylase, and 5-hydroxytryptamine). The density of the nerve fiber loaded with neuropeptides and some of their specific receptors within the male genital tract appeared to differ in various mammalian species. Apart from these, evidence of variation in age, season, and intraspecies differences were identified as notable factors of influence in peptidergic nerve fiber distribution. A summary on the overview of the neuropeptides discussed here is provided in Table 1, highlighting the various mammalian species in which the neuropeptide components of their male genitalia have been studied, their anatomical locations, and physiological roles. Despite vast efforts in the localization of the peptides using immunohistochemistry protocols, there is still a need for comprehensive neuropeptide receptor profiling, mechanistic probing of neuropeptide action using pharmacological-based evidence, and sexual hormonal interplay, as these are crucial to further unravel the functional relevance of neuropeptides in the male genital tract. Other mechanisms that could prove useful in profiling the neuropeptides of the male genitalia is the untargeted neuropeptidomics mass spectrometry, as this has the capacity of defining the structural features of several thousands of neuropeptide prevalence in biological systems. In spite of this upsurge in documentation of neuropeptides in mammalian male genital tract, there is still a long way to go, as there are several mammals, primates, and some uncommon rodents especially in Africa, as well as numerous non-mammalian species with neuropeptidergic components that remain unreported, and these knowledge gaps need to be filled in the near future.

## Author Contributions

JOO conceptualized the manuscript. JOO and IAA contributed to writing (original draft and review & editing) and making the table in this article, and approved its submission for publication. Both authors approved the submitted final version.

## Conflict of Interest

The authors declare that the research was conducted in the absence of any commercial or financial relationships that could be construed as a potential conflict of interest.

## Publisher's Note

All claims expressed in this article are solely those of the authors and do not necessarily represent those of their affiliated organizations, or those of the publisher, the editors and the reviewers. Any product that may be evaluated in this article, or claim that may be made by its manufacturer, is not guaranteed or endorsed by the publisher.
